# En Bloc Resection of Coracoid Base Osteoid Osteoma in a Child: An Extremely Rare Case

**DOI:** 10.5812/ircmj.9534

**Published:** 2013-11-05

**Authors:** Mohammad Gharahdaghi, Hassan Rahimi Shorin, Ali Parsa, Maryam Assadian

**Affiliations:** 1 Department of Orthopedic Surgery, Mashhad University of Medical Sciences, Mashhad, IR Iran; 2 Department of Orthopedic Surgery, Zahedan University of Medical Sciences, Zahedan, IR Iran

**Keywords:** Osteoid Osteoma, Child, Resection

## Abstract

**Background:**

Osteoid osteomas account for approximately 2- 3% of all primary bone neoplasm's sampled for biopsy. 50% of all of them occur in the long bones of the lower extremities, but osteoid osteoma of the coracoid process is rare. We have found only nine previously reported cases of coracoid osteoid osteoma in the international literature. We report a child with coracoid base osteoid osteoma.

**Case presentation:**

A twelve-years-old boy with a persistent pain at the right shoulder since 9 months ago, were evaluated. His pain relatively responds to non-steroidal anti-inflammatory drugs (NSAIDs). Imaging studies showed foci of sclerosis at right coracoid base with increased uptake on the Technetium-99m study. Because proximity to the neurovascular bundle we couldn't use radiofrequency ablation technique, so the patient underwent open surgery with a mini- deltopectoral approach and two level osteotomies ; one proximal to coracoid tip and the other at the base of coracoid just distal to subcoracoidphyseal line. Then a segment of coracoid resected. Tip of coracoid securely reattached with a screw.

**Conclusions:**

To our knowledge in the literature up to now there are only nine reports of coracoid osteoid osteoma. These cases were treated with different approaches and different techniques. We used en-bloc resection via mini-anterior approach. Our patient 30 months after surgery (October 2012) have full range of motion and became pain free since wound healing with normal control imaging.Definite diagnosis of osteoid osteoma in the uncommon sites may be delayed. En bloc resection of tumor with two osteotomies by an anterior approach has been limited soft tissue injury and is a reliable method of treatment.

## 1. Introduction

Osteoid osteomas are solitary, benign, painful, lesions of the bone (1). Osteoid osteomas were described as a distinct entity by Jaffe in 1935(2). These tumors consist of a welldemarcatedosteoblastic mass called a nidus that is surrounded by a distinct zone of reactive bone sclerosis. The zone of sclerosis represents a secondary reversible change that gradually disappears after removal of the nidus(3). Osteoid osteomas account for approximately 2- 3% of all primary bone neoplasms sampled for biopsy (4). 50% of all of them occur in the long bones of the lower extremities(3), but osteoid osteoma of the scapula and its localization to the coracoid process is rare(5).We have found only nine previously reported cases of coracoid osteoid osteoma in the international literature and reported management of osteoid osteoma in an extremely rare anatomic site.

## 2. Case Report

A twelve-years-old boy with a good health condition and without any trauma began toexperience pain at the right shoulder. The pain was mild at the beginning and graduallyincreased. At the time of referring (April 2010) to our clinic in Mashhad University of Medical Science,Iran; he had a 9 months history of right shoulder pain. This pain became persistent with night acceleration and relatively responds to non-steroidalanti-inflammatory drugs (NSAIDs). On further examination the pain located in front of right shoulder with no obvious tender or swollen area. There was no regional lymph node enlargement. True AP X-ray only showed limited sclerosis at right coracoid base.

**Figure 1.**
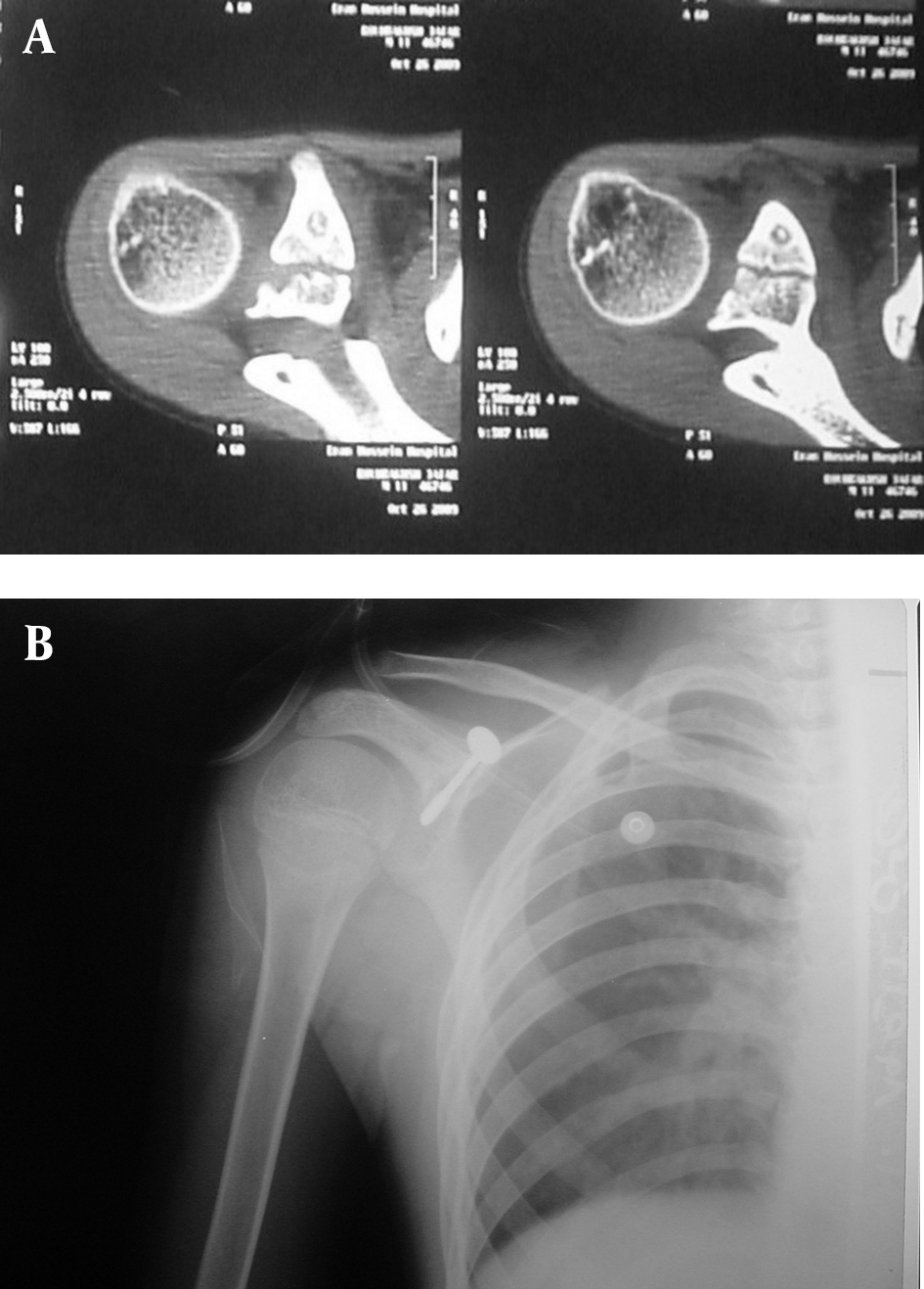
A) Apparent Nidus in Semicoronal CT-s Images, B) Screw Fixation of Coracoid After Osteotomies

Complete blood count, Erythrocyte sedimentation rate and C-reactive protein were allnormal.Technetium-99m bone scan showed increase uptake on the right coracoid process,then CT-scan revealed a wellcircumscribed area representing the nidus above the sub-coracoidcenterof ossification (Figure 1 A).Because proximity to the neurovascular bundle we couldn't use radiofrequency ablation technique, so the patient underwent open surgery with amini deltopectoral approach, The coracoids was exposed with preservation of all attachments of pectoralis minor, conjoined tendon, coracoacromial and coracoclavicular ligaments; Then two osteotomies were performed; first 7-8 mm proximal to coracoid tip and the second osteotomy the other at the base of coracoid just distal to subcoracoidphyseal line, a segment of coracoid about 2 cm long resected which contains lesion and its surrounding sclerosis; at gross morphology the cone shape section had a grayish nidus. Intra operative radiograph of resected fragment of bone shows a small nidus. 

**Figure 2.**
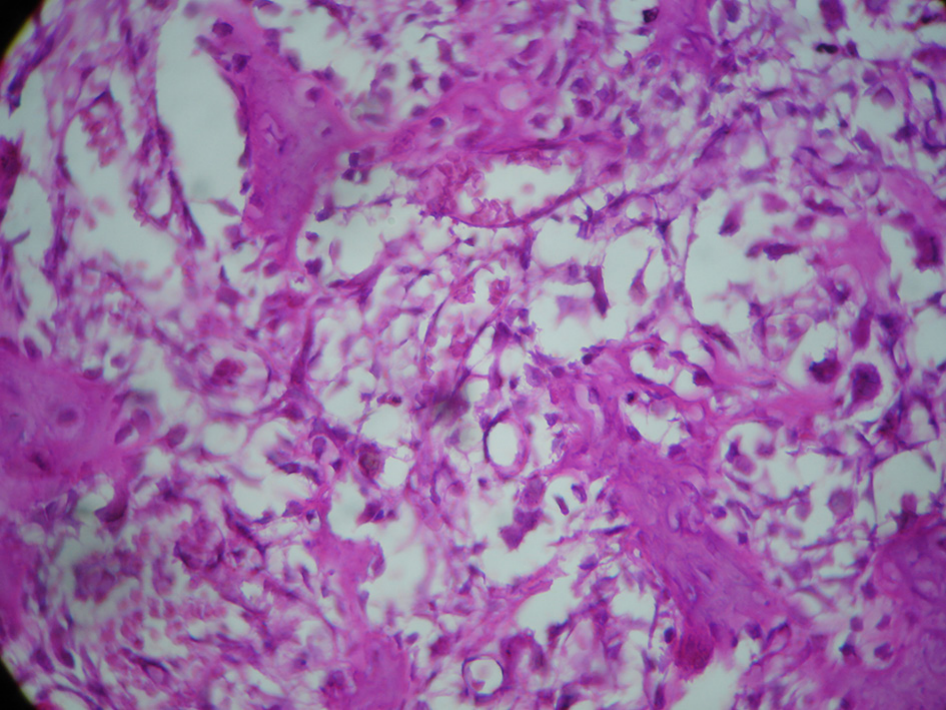
Higher Power View of Shows Prominent Plump Osteoblasts Loosely Occupying Intertrabecular Spaces

Tip of coracoid securely re-attached to its anatomic base and fixed with a 36 mm length, 4 mm cancellous screw and the incision was repaired in layers (Figure 1 B).The shoulder supported in a sling for 4 weeks. Histological examination revealed a fibro vascular tissue with osteoid formation and prominent plump osteoblasts compatible with osteoid osteoma (Figure 2). 

## 3. Discussion

Typically patients with osteoid osteomaare being presented with a long duration of dull pain in the shoulder region due to several cause of anterior shoulder pain and rarity of osteoid osteoma; thus it is inevitable to have delay in the diagnosis. Most of the tumors are intracortical, with the nidus appearing as a radiolucent lesion. This nidus rarely exceeds 1 cm in diameter but may be as large as 2 cm (6). Minimal or absent perilesional sclerosis is a common feature of osteoid osteomas that are located near the end of bone (juxta-articular), intramedullary and in subperiosteal lesions (3). Technetium-99m bone scan nearly always demonstrates an intense focal increase of uptake in the nidus(7). After the general area of the lesion has been localized with bone scan, CT demonstrates the area representing the nidus better. In MRI the nidus does not show high enough level of signal intensity in T1-weighted scan and its signal level in T2-weighted scan is not reliable either (8). Some tumors may even spontaneously regress (9, 10). Surgical excision has proved effective in eradicating the pain-producing nidus(11), Preferred treatment is en bloc excision after precise localization of the nidus(12, 13).

The first case of osteoid osteoma of the coracoid process was reported by Kaempfee in 1994, he exposed the nidus via the posterior approach and curetted it. Ogose et al. managed two cases of coracoid osteoid osteoma with resection and curettage, but without reference to their approach in the report (14). In 2001 Akpinar and Gracia in two distinct papers reported another two cases which treat them through an anterior approach excision (8, 15). Angius from Mayo clinic in 2007 reported two patients with infraclavicular brachial plexopathy secondary to coracoid osteoid osteoma, with excellent long-term outcomes after tumor removal (16). In this case we use en-bloc resection via a mini anterior approach. The postoperative X-rays werereviewed to assess the bone union andpain.Active and passive ranges of motion were evaluated in treated shoulder. Our patient 30 months after surgery (October 2012) hadfull range of motion andhe didn’t feel any pain after healing.

lack of awareness and ability to mimic other disorders clinically leads to diagnostic and therapeutic delays in patients with persistent shoulder pain , high level of suspicious has to be maintained in order to diagnose osteoid osteoma in thethe uncommon site;. En bloc resection of tumor with two osteotomies by an anterior approach causes small amount ofsoft tissue injury and is a reliable method of treatment.
